# Surveying the genetic factors effect to lack of palmaris longus muscle's tendon and prevalence of absence in the inhabitants of Eastern Azerbaijan

**DOI:** 10.1186/1753-6561-6-S4-P34

**Published:** 2012-07-09

**Authors:** A Hashemiaghdam, A Iranmehr, F Abolhassani, A Meysamie, L Ghadakchi

**Affiliations:** 1 Faculty of Medicine, Tehran University of Medical Sciences, Iran; 2 Department of Public Health, Faculty of Medicine, Tehran University of Medical Sciences, Iran; 3 Department of Anatomical Sciences, Faculty of Medicine, Tehran University of Medical Sciences, Iran; 4 Faculty of Medicine, Tabriz University of Medical Sciences, Iran

## Introduction

Palmaris longus (PL) is phylogenetically classified as a retrogressive muscle with a long tendon. The length and location of the PL tendon makes it very useful in reconstructive surgery. Agenesis of PL tendon shows strong racial variations. Past studies show absence of 2.8% to 25% depending on ethnicity. Chinese have a low absence rate of 6.0% whilst Caucasian subjects had a high absence rate of 25%. The aim of this study is to determine the incidence of unilateral and bilateral absence of PL tendon for Eastern Azerbaijan region of Iran and surveying the genetic factors and environmental effects to lack of PL tendon.

## Methods

Each subject’s data are gathered in specific form. Individuals with history of injury or abnormality of the upper extremities were excluded. Writing and skill hand dominance and job type were recorded. The exercise was conducted with two different methods of assessment; standard test (Schaeffer’s test), Thompson’s test. In Schaeffer’s test, volunteers were made to steady their forearm at 90° before opposing the thumb to the little finger with the wrist partially flexed. In Thompson’s test, a fist was made followed by flexing the wrist against resistance with the thumb flexed over the fingers.

## Results

Our study sample included 1247 subjects (50.6% males, 49.4% females). The prevalence of absence was 24.4% (17.2% unilaterally absent, 7.1% bilaterally absent) (p<0.0001; Chi-square test). The absence rate in male subjects (19.8%) was lower than female subjects (29.1%). In subjects with no absence in right hand, the absence in the left hand was 11.0% and subjects with no absence in their left hand, the absence in the right hand was 9.6% (p<0.0001; chi-square test). In cases with absence in left hand, the rate of bilaterally absence was 43.6% also same rate in subjects with absence in the right hand was 47.3% (p<0.0001; chi-square test).

## Conclusions

Our data suggest that the prevalence of absence of Palmaris Longus tendon in our study sample in Iran has a high value of absence, similar to Caucasian samples. The absence rate was lower in male and right handed subjects.

